# Ionic Transportation and Dielectric Properties of YF_3_:Eu^3+^ Nanocrystals

**DOI:** 10.3390/nano8120995

**Published:** 2018-12-01

**Authors:** Xiaoyan Cui, Tingjing Hu, Jingshu Wang, Junkai Zhang, Xin Zhong, Yanli Chen, Xuefei Li, Jinghai Yang, Chunxiao Gao

**Affiliations:** 1 Key Laboratory of Functional Materials Physics and Chemistry of the Ministry of Education, National Demonstration Center for Experimental Physics Education, Jilin Normal University, Siping 136000, China; xycuimail@163.com (X.C.); jingshuwang126@126.com (J.W.); junkaizhang126@126.com (J.Z.); zhongxin@calypso.cn (X.Z.); chenqian358@126.com (Y.C.); xuefeili163@163.com (X.L.); jhyang1@jlnu.edu.cn (J.Y.); 2 State Key Laboratory of Superhard Materials, Jilin University, Changchun 130012, China; chunxiaogao126@126.com

**Keywords:** nanocrystals, ionic transportation, dielectric behavior, permittivity

## Abstract

The ionic transportation and dielectric properties of YF_3_:Eu^3+^ nanocrystals are investigated by AC impedance spectroscopy. The ion diffusion coefficient and conductivity increase along with the doping concentration and reach their highest values at 4% of Eu^3+^. The difference of ionic radius between Eu^3+^ and Y^3+^ leads to the structural disorder and lattice strain, which deduces the increase of the ion diffusion coefficient and conductivity before 4% Eu^3+^ doping; then the interaction of the neighboring doping ions is dominated, which results in the difficulty of ion migration and decreases of the ion diffusion coefficient and conductivity. The strong dispersion of the permittivity in the low frequency region indicates that the charge carrier transport mechanism is the ion hopping in the system. The low-frequency hopping dispersion is affected by an interfacial polarization, which exhibits a Maxwell-Wagner relaxation process, and its loss peak shifts to higher frequency with the ionic conductivity increasing.

## 1. Introduction

Rare-earth fluoride nanomaterials have attracted a great deal of interest due to their unique physical and chemical properties and potential applications in luminescence, optoelectronic, down/up conversion devices, etc. [1,2,3,4,5,6]. Among the rare earth fluorides, YF_3_ is an important material and has widespread potential applications in optical telecommunication, phosphors, down/up conversion luminescent devices, solid-state batteries, sensors [6,7,8,9,10,11,12,13,14], etc. Due to the closed ionic radius and the same valence, the Y^3+^ can be easily replaced by other rare earth ions [15,16]. Moreover, YF_3_ has low refractive index and wide bandgap [17,18]. Therefore, YF_3_ is an attractive host material for lanthanide-doped phosphors. At the same time, due to its high ionic conduction, YF_3_ is a prospective material used as a solid electrolyte of solid-state electrochemical devices.

As the phosphor host and solid electrolyte material, the ionic conductivity and dielectric properties of YF_3_ nanocrystals are important subjects. Many investigations have been focused on the structure, synthesis and optical properties of YF_3_ nanocrystals [8,9,10,11,19,20,21]. However, few reports have been conducted on the electrical and dielectric properties of YF_3_. Only Sathyamoorthy et al. have investigated the AC conduction of YF_3_ thin film, whose structure is amorphous [22]. A thorough study on the carrier transport and dielectric properties of YF_3_ nanocrystals is necessary. In this letter, the ionic transportation and dielectric properties of YF_3_:Eu^3+^ nanocrystals are investigated by AC impedance spectroscopy. The ionic diffusion, ionic conductivity and complex permittivity are discussed with various Eu^3+^ doping concentrations.

## 2. Materials and Methods

The synthetic process of YF_3_:Eu^3+^ nanocrystal is similar to that of alkaline earth fluorides [23,24,25,26]. The mixed solution of oleic acid, ethanol and ammonia was stirred for 10 min; then 3.0641 g Y(NO_3_)_3_·6H_2_O and 0.7402 g NH_4_F were added and stirred thoroughly. The above solution was put into an autoclave and then reacted for 24 h at 190 °C. The final production was obtained through centrifugation, washing and drying. The Eu^3+^-doping samples was obtained by replacing Y(NO_3_)_3_ with a certain amount of Eu(NO_3_)_3_ and the Eu^3+^ doping concentrations are 2, 4, 6, 8, 10 mol% respectively. The morphology and particle size of sample were obtained by transmission electron microscope (TEM). The structure and component of sample were obtained by X-ray diffraction (XRD) and energy dispersive spectrometer (EDS). The detailed measurement process of AC impedance spectroscopy was described in our previous works [27,28]. The spectra were measured by parallel plate electrode. The copper sheet was used as electrode, which is an ions’ blocking material. The sample chamber is a cylinder (ø6 mm × 1 mm). The amplitude of AC voltage needs to be selected according to the sample. Though our testing, the appropriate input voltage for this sample is 1 V. The frequency is 0.01–10^7^ HZ.

## 3. Results and Discussion

**Figure 1:**
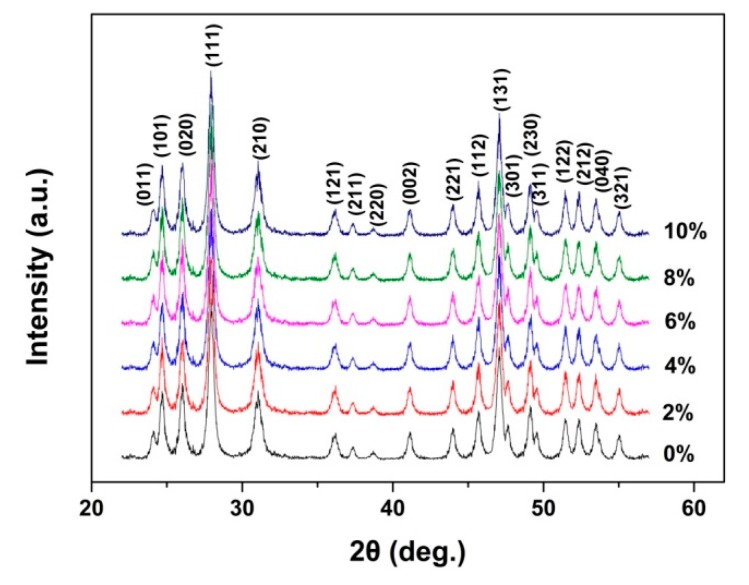
The XRD spectrum of YF_3_:Eu^3+^ nanocrystals.

**Figure 2:**
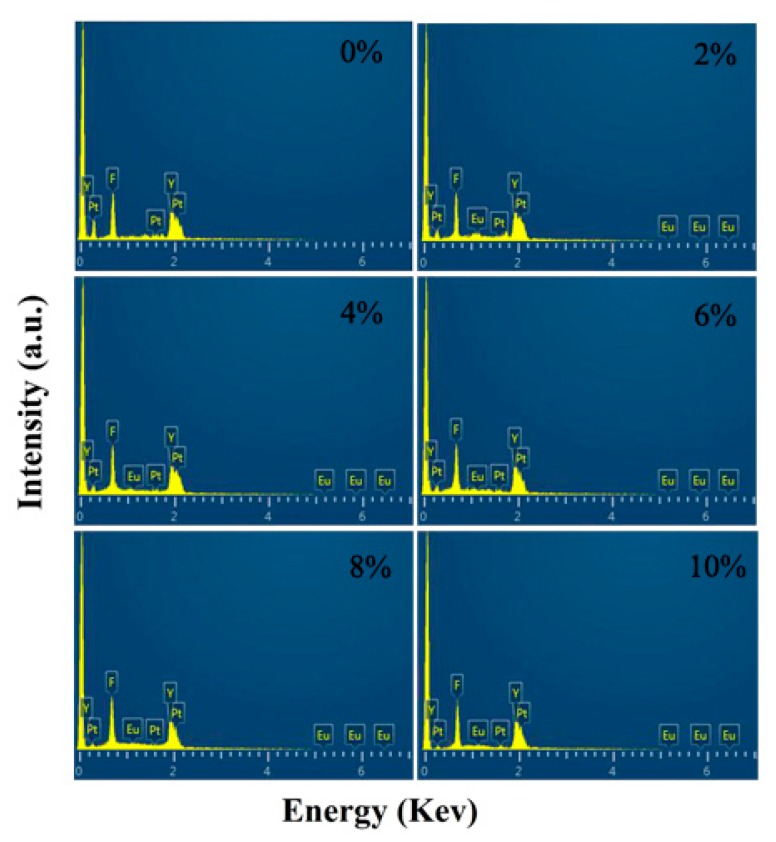
The EDS spectrum of YF_3_:Eu^3+^ nanocrystals.

**Figure 3:**
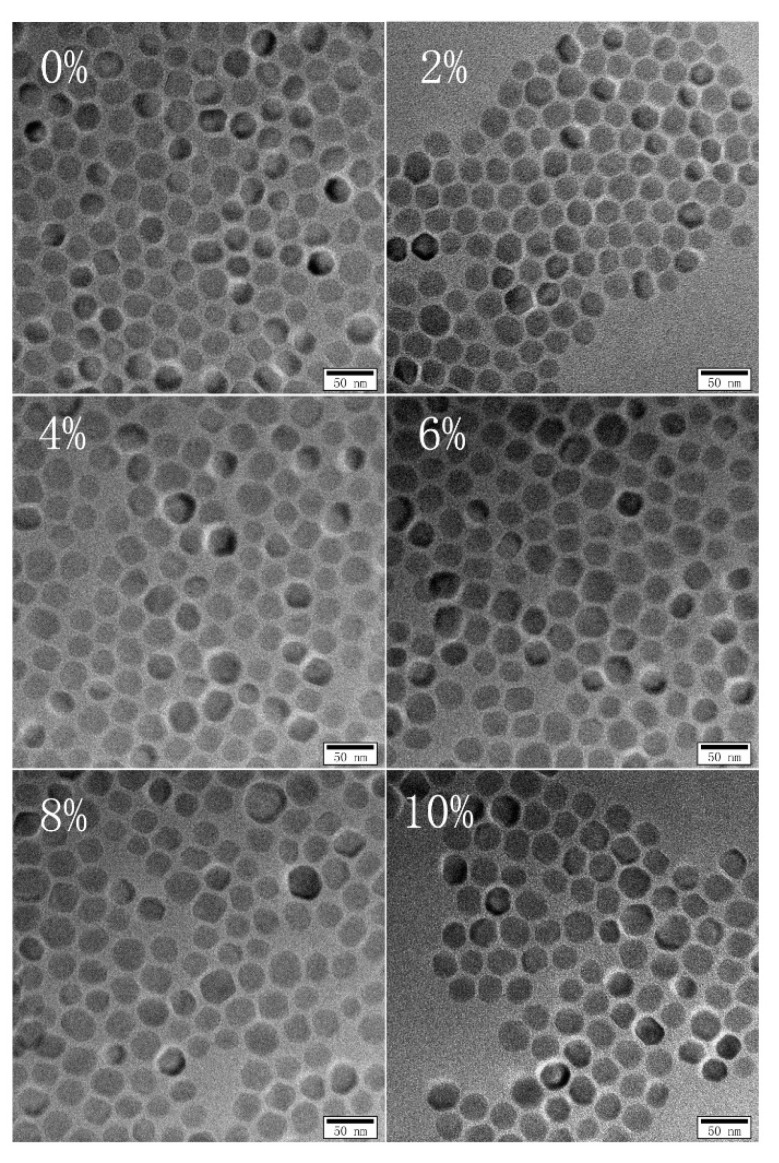

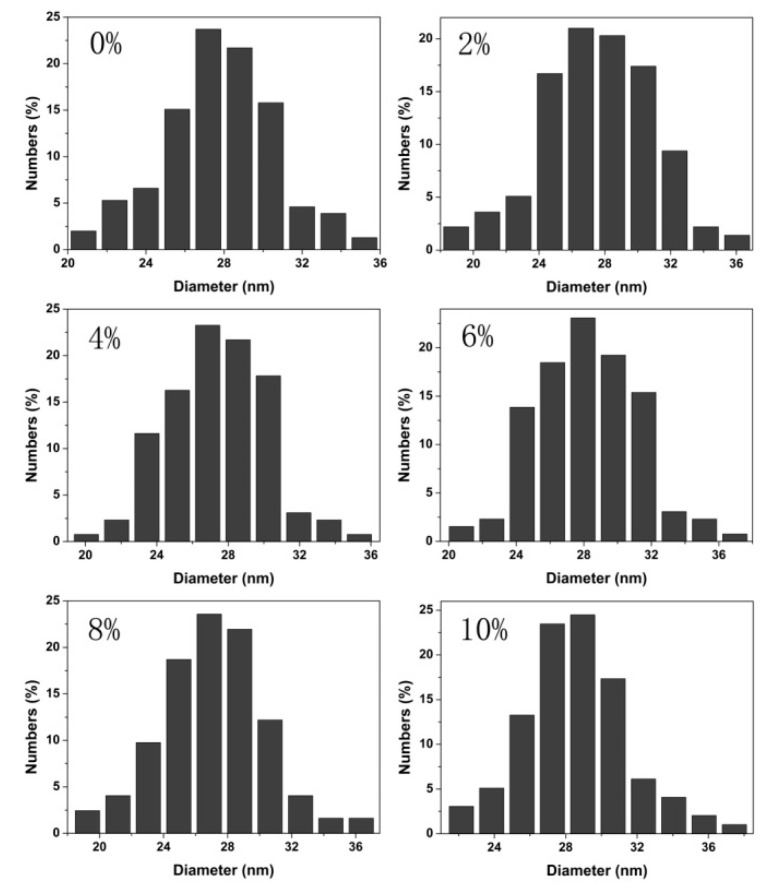
The TEM photo and the size distribution histogram of YF_3_:Eu^3+^ nanocrystals.

The structure, component and morphology of YF_3_ with different Eu^3+^-doping concentrations were investigated by XRD, EDS and TEM as shown in Figure 1, Figure 2 and Figure 3. It can be seen that all the XRD spectra can be indexed as orthorhombic YF_3_ structure (JCPDS Card No. 74-0911), and the existence of Eu peak in the EDS spectra proves that the Eu^3+^ ion successfully replaces Y^3+^ in the Eu^3+^-doping samples. The TEM results show that all the samples are well crystallized and all of their particle sizes are about 28 ± 4 nm.

**Figure 4:**
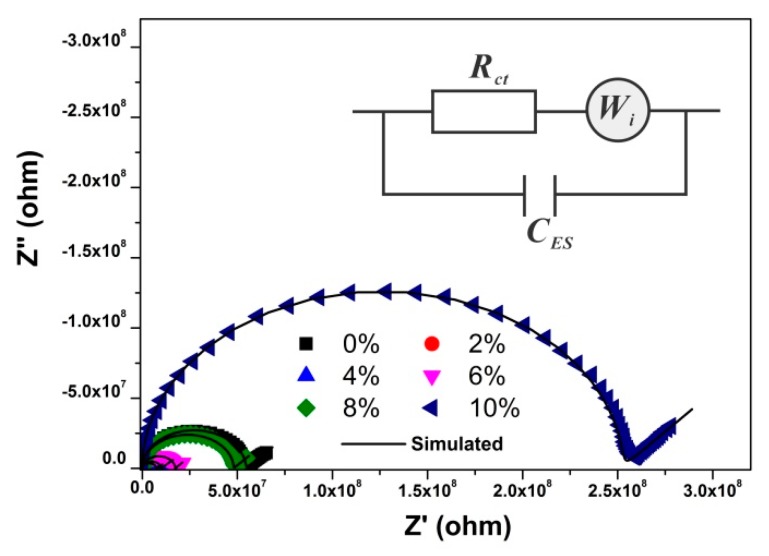
The impedance plots of YF_3_:Eu^3+^ nanocrystals. The DC polarization voltage for the impedance plots (V_DC_) is 0 V. The insert is the equivalent circuits, *R_ct_* is the ion transfer resistance, *W_i_* is Warburg element, *C_ES_* is the capacitance between the sample and electrode.

The impedance plots of YF_3_ with different Eu^3+^-doping concentrations are shown in Figure 4. It shows that all the spectra display a semicircle and a straight line. To obtain the transport parameters, the impedance spectra are usually analyzed by the equivalent circuits. The straight line indicates the existence of the Warburg diffusion process [29]; therefore, a Warburg element was added into the equivalent circuit. The equivalent circuit we used was shown in the insert of Figure 4 and the fitting parameters for the impedance plots were listed in Table 1. The good agreements of the impedance spectra and the simulate plots indicate that the equivalent circuit is appropriate.

**Figure 5:**
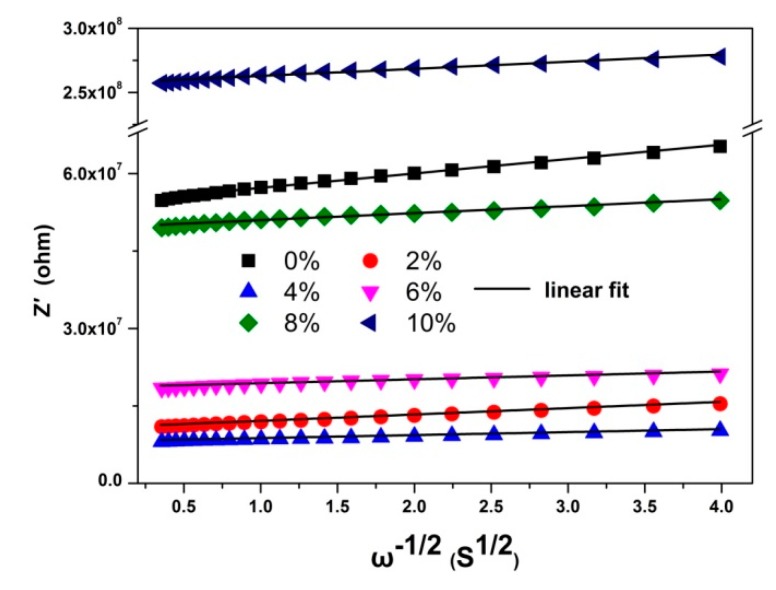
The *Z′*~*ω^-1/2^* plots of YF_3_:Eu^3+^ nanocrystals at low frequencies.

To the Warburg diffusion process, the real impedance part (*Z^′^*) in the low frequency region can be expressed as [29,30,31]:

(1) $$\;Z^{\prime }=Z_{0}^{\prime }+\sigma \omega^{-1/2}$$

where *σ* is Warburg coefficient, $Z_{0}^{\prime }$ is constant and *ω* is frequency. By linear fitting the plots of *Z′*~*ω^-1/2^* [as shown in Figure 5], the Warburg coefficient *σ* was obtained.

**Figure 6:**
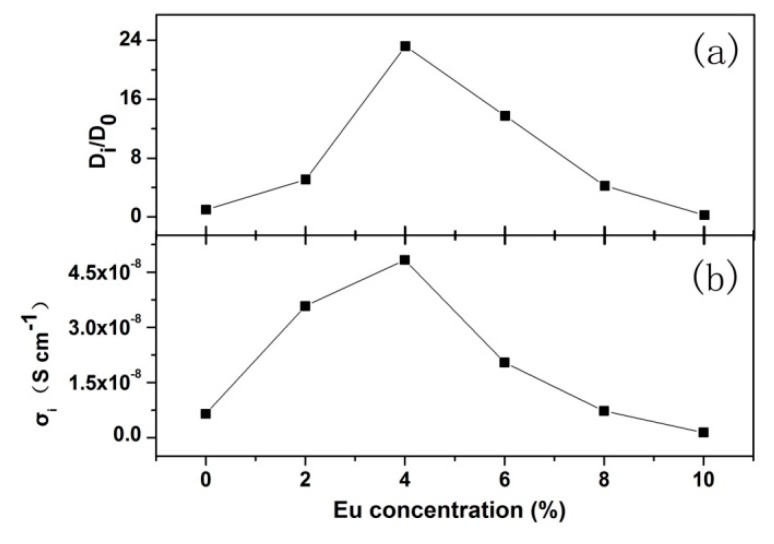
The variation of the ionic diffusion coefficient (**a**) and conductivity (**b**) with the Eu-doping concentration.

The diffusion coefficient of F^-^ ion can be gained by the following equation,

(2) $$D_{i}=0.5{\left(\frac{RT}{AF^{2}\sigma C}\right)}^{2}$$

where *R* is the ideal gas constant, *T* is temperature, *F* is the Faraday constant, *C* is the molar concentration of ions. Let *D_0_* be the F^−^ ion diffusion coefficient of un-doped YF_3_, then the *D_i_/D_0_* of different Eu-doping concentration can be gained and was shown in Figure 6a.

By simulating the spectra with the equivalent circuit, the ion transfer resistances (*R_ct_*) of YF_3_ with different Eu-doping concentrations are obtained. The ionic conductivity *σ_i_* can be gained by the following formula:

(3) $$\sigma_{i}=\frac{d}{R_{ct}A}$$

where *d* is the sample thickness, and *A* is the electrode area. The Eu-doping concentration dependence of the ionic conductivity was shown in Figure 6b. 

It can be seen that the ion diffusion coefficient and conductivity increase with the doping concentration until they reach 4% and then decrease. The changing mechanism of the two parameters can be interpreted as the following: the Eu^3+^ ionic radius is larger than that of Y^3+^; while the size mismatch will lead to the structural disorder and lattice strain [1], which finally deduces the increase of the ion diffusion coefficient and conductivity. When the doping concentration is larger than 4%, the interaction of the neighboring doping ions emerges due to the decrease of distance between them [32], which results in the difficulty of ion migration and finally causes the decreases of the ion diffusion coefficient and conductivity.

**Figure 7:**
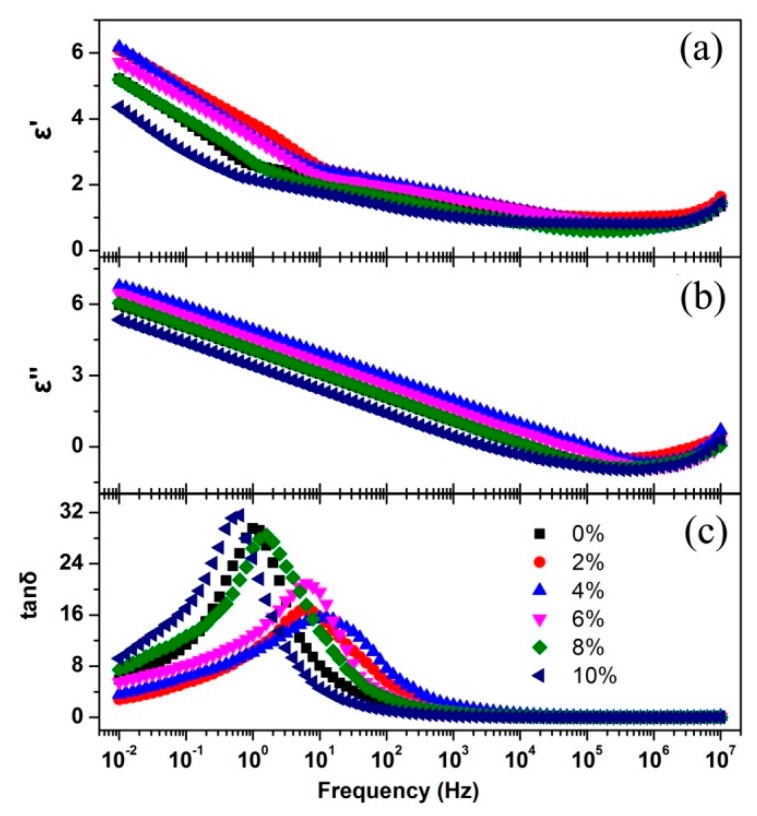
The frequency dependence of *ε′* (**a**), *ε″* (**b**) and tan*δ* (**c**) with different Eu^3+^ doping concentration.

To further explore the carrier transport mechanism of YF_3_:Eu^3+^, its dielectric properties were investigated. The frequency dependence of the permittivity real part (*ε′*), imaginary part (*ε″*) and the dielectric loss tangent (tan*δ*) with different Eu^3+^ doping concentration were shown in Figure 7.

It can be seen that with frequency increasing *ε′* decreases sharply, then decreases slowly, and finally increases. *ε″* decreases almost linearly with frequency increasing and then increases. The strong dispersion of the permittivity in the low frequency region indicates the charge carrier transport mechanism is the ion hopping in the system [33]. Ionic hopping behavior occurs as follows: under the action of electric field, one F^-^ ion moves to a more advantageous site (vacancy or interstitial position), then a vacancy is formed at this position and becomes an advantageous site to its surrounding atoms and so on. From Figure 7c, it can be seen that all the dielectric loss tangent plots show well-defined peaks in the frequency region of 0.1–100 HZ, which indicates the existence of the interfacial polarization between the grain boundaries in all of the samples. This loss peak distribution is in agreement with the Maxwell-Wagner relaxation model [34]. It can be deduced that the hopping dispersion in this region is affected by this interfacial polarization, and this phenomenon is similar to the result obtained from AC conduction investigation on YF_3_ thin film conducted by Sathyamoorthy et al. [22]. From Figure 7, it can be concluded that with the increasing of ionic conductivity, the *ε′* and *ε″* increase in the low frequency region and the loss peak of tan*δ* plot shifts to higher frequency. Before 4% Eu^3+^ doping, the structural disorder and lattice strain increase with the doping concentration increasing, which are beneficial for the ion migration, and finally result in the ion hopping becoming easier and the interfacial polarization relaxation frequency increasing. When the doping concentration is larger than 4%, the interaction of the neighboring doping ions is strengthened and its effect is dominated; the scattering to ions and dipoles enhances; which finally makes the ion hopping more difficult and makes the interfacial polarization relaxation frequency decrease.

## 4. Conclusions

The structure, component and morphology of YF_3_:Eu^3+^ nanocrystals were measured by XRD, EDS and TEM. The ionic transportation and dielectric properties of YF_3_:Eu^3+^ nanocrystals are investigated by AC impedance spectroscopy. The ion diffusion coefficient and conductivity increase with the doping concentration increasing and reach their highest values at 4% of Eu^3+^. The difference of ionic radius between Eu^3+^ and Y^3+^ leads to the structural disorder and lattice strain, which deduces the increase of the ion diffusion coefficient and conductivity before 4% Eu^3+^ doping. Afterwards, the interaction of the neighboring doping ions is dominated, which results in the difficulty of ion migration and the decreases of the ion diffusion coefficient and conductivity. The strong dispersion of the permittivity in the low frequency region indicates that the charge carrier transport mechanism is the ion hopping in the system. The low-frequency hopping dispersion is affected by an interfacial polarization, which exhibits a Maxwell-Wagner relaxation process and its loss peak shifts to higher frequency with the ionic conductivity increasing. We have conducted a thorough study on the ionic transportation and dielectric properties of YF_3_ nanocrystals with different Eu^3+^ doping concentration and it is hoped that the design of YF_3_-based devices could benefit from this investigation.

## Figures and Tables

**Table 1:** The fitting parameters of the equivalent circuit for the impedance plots.

Eu Concentration	*R_ct_* (Ω)	*C_ES_* (F)	*W_i_-R* (Ω)	*W_i_-P*	*W_i_-T* (S)
0%	5.44 × 10^7^	7.55 × 10^−12^	8	0.260	7.50 × 10^−12^
2%	9.90 × 10^6^	6.55 × 10^−12^	7	0.246	6.50 × 10^−12^
4%	7.32 × 10^6^	5.55 × 10^−12^	6	0.235	5.55 × 10^−12^
6%	1.73 × 10^7^	7.95 × 10^−12^	2	0.265	9.95 × 10^−12^
8%	4.89 × 10^7^	5.05 × 10^−12^	3	0.265	5.50 × 10^−12^
10%	2.52 × 10^8^	5.05 × 10^−12^	10	0.270	5.05 × 10^−12^
